# Predictors of treatment benefits after enhanced external counterpulsation in patients with refractory angina pectoris

**DOI:** 10.1002/clc.23516

**Published:** 2021-01-05

**Authors:** Eline Wu, Jan Mårtensson, Liyew Desta, Anders Broström

**Affiliations:** ^1^ School of Health and Welfare Jönköping University Jönköping Sweden; ^2^ Heart and Vascular Theme Karolinska University Hospital Stockholm Sweden; ^3^ Department of Nursing, School of Health and Welfare Jönköping University Jönköping Sweden; ^4^ Department of Medicine, Division of Cardiology Karolinska Institute Stockholm Sweden; ^5^ Department of Clinical Neurophysiology Linköping University Hospital Linköping Sweden

## Abstract

**Background:**

Enhanced external counterpulsation (EECP) is a noninvasive treatment that can decrease limiting symptoms in patients with refractory angina pectoris (RAP). Identifying responders to EECP is important as EECP is not widely available and relatively time intensive.

**Hypothesis:**

The effect of EECP treatment on physical capacity in patients with RAP can be predicted from baseline patient characteristics and clinical factors.

**Methods:**

This explorative study includes all patients from a cardiology clinic who had finished one EECP treatment and a 6 min walk test pre and post EECP. Clinical data, including Canadian Cardiovascular Society (CCS) classification and left ventricular ejection fraction (LVEF), were assessed before treatment. If patients increased their 6 min walking distance (6MWD) by 10% post EECP, they were considered responders.

**Results:**

Of the 119 patients (men = 97, 40–91 years), 49 (41.2%) were responders. Multinomial regression analysis showed that functional status (i.e., CCS class ≥3) (OR 3.10, 95% CI 1.12–8.57), LVEF <50% (OR 2.82, 95% CI 1.02–7.80), and prior performed revascularization (i.e., ≤ 1 type of intervention) (OR 2.77, 95% CI 1.06–7.20) were predictors of response to EECP (p < .05, Accuracy 63.6%). Traditional risk factors (e.g., gender, smoking, and comorbidities) did not predict response.

**Conclusions:**

EECP treatment should be considered preferentially for patients that have a greater functional impairment, evidence of systolic left ventricular dysfunction, and exposure to fewer types of revascularization, either PCI or CABG. Improvement in 6MWD post EECP could imply improvement in physical capacity, which is a likely contributor to improved well‐being among patients with RAP.

## 1 INTRODUCTION

Patients with refractory angina pectoris (RAP) experience chronic symptoms, characterized by chest pain, in the setting of coronary artery disease (CAD), which is uncontrolled despite optimized therapy.^1^, ^2^ In the European guidelines CAD is categorized as either acute coronary syndrome or chronic coronary syndrome, with RAP in the latter category.^3^ The prevalence of RAP is 5–10% in stable CAD,^4^, ^5^ with a yearly incidence of 50 000–100 000 in the US and 30 000–50 000 in Europe.^4^, ^6^ This heterogeneous group remains significantly limited in daily life despite revascularization and optimized medication.^2^ Many are not considered for further revascularization when deteriorating in angina symptoms due to unsuitable anatomy, a negative risk–benefit profile, or advanced age.^4^ Consequently, many adjust their activities to avoid cardiac events,^7^ which causes a sedentary lifestyle,^3^ and a decreased health‐related quality of life (HRQoL).^8^, ^9^ To improve these patients' daily life other treatment options are needed.

In 1995, the US Food and Drug Administration (FDA) approved Enhanced External Counterpulsation (EECP)^10^ for patients with RAP^3^ (for mechanisms and protocol see ^9^, ^11^, ^12^, ^13^) Parameters like functional class, changes in consumption of short‐acting nitrates and N‐terminal pro‐brain natriuretic peptide (NT‐proBNP) has been used to assess clinical effects.^14^, ^15^, ^16^, ^17^, ^18^ A recent study^19^ showed a significantly improved walking distance, as measured by 6 min walk test (6MWT), after EECP treatment. The use of 6MWT can be relevant since it is a simple and cost‐effective way to assess physical activity^20^, but there is limited evidence regarding its prognostic ability, since previous studies have been mainly in patients with heart failure.^21^, ^22^ Interpreting a change in walking distance requires knowledge of the difference perceived as important to the patient (i.e., minimal clinically important difference, MCID).^23^ Unfortunately, the literature does not provide MCID for 6MWD for patients with RAP or in an EECP context, but a change of more than 50 meters is believed to be clinically significant in most disease conditions.^23^ However, reporting improvement in meters can give misleading results.^22^ Instead, improvement based on proportion may be a better alternative.

EECP has beneficial effects and has been used as an additional alternative when conventional therapy has not been successful to adequate symptom control, but 15–30% of treated patients do not experience neither subjective nor objective clinical improvement.^12^, ^16^, ^24^ Identification of potential responders to EECP before treatment initiation could be reasonable as the treatment is not widely available, it is relatively time and resource intensive and a significant proportion of patients do not experience benefit. Therefore, this study aimed to identify patients who respond well to EECP by using 6MWD as an outcome measure and to identify predictors of response after treatment.

## 2 METHODS

### 2.1 Study design and study population

This study was conducted by exploring a database of all 141 patients who had undergone one course of EECP treatment at a cardiology clinic in a metropolitan university hospital between 2005 and 2019. Before EECP, all the patients were deemed to be noncandidates for further invasive treatment by cardiologist. The course of EECP was given according to a standard clinical protocol and divided into 35 1 h daily sessions over 7 to 9 weeks. Two devices were used—TS4 in early years and the later with Luminair (Vasomedical Inc., New York). Both consisted of an air compressor, a computer module, a set of cuffs, and a treatment bench. Pneumatic cuffs (connected by air hoses to the air compressor) were wrapped around the calves and lower thighs including the buttocks. The pressure (240–260 mmHg) in the cuffs was applied from the calves up to the buttocks in a sequence that was synchronized to the patient's cardiac cycle through a one‐lead electrocardiogram.^12^ Inclusion criteria included full completion of one EECP course and two 6MWTs (one before and one directly after the EECP course). Twenty‐two patients lacked two 6MWTs, which resulted in a final sample of 119 patients.

### 2.2 Study variables and data collection

All data were collected by clinical cardiac nurses with several years of experience in providing EECP treatment at the clinic. Sociodemographic information, medical history, clinical data, and laboratory data were derived from medical records. The patients' functional class was assessed by either a cardiac nurse or a physician using the Canadian Cardiovascular Societies (CCS) classification (i.e., class 1–4), a well‐known tool used to classify symptoms in relation to different levels of physical activity.^25^ Data on left ventricular function (LVEF) were derived from either an echocardiography or a stress cardiac imaging examination, which was performed before EECP treatment according to clinical routine care. The treatment aims to create an enhanced diastolic augmentation during cardiac diastole and the efficiency of counterpulsation is measured as the ratio between arterial diastolic to systolic pressures (i.e., D/S ratio).^12^, ^13^ The D/S ratio was monitored using standard finger plethysmography.^13^ The D/S ratios were recorded as an average during each EECP session by the cardiac nurse.

The 6MWT was performed pre and post EECP as recommended by the American Thoracic Society (i.e., walking alone continuously back and forth for 6 min on a hard‐flat surface and marked distance in a hospital hallway).^26^ Patients were encouraged with standardized phrases to carry on the walk test until reaching maximal exertion and were monitored for any pain or discomfort.^27^ Perceived exertion and standard pain or chest discomfort were rated by the patient according to the Borg scales before and after the walk test.^26^ The chosen outcome measure was improvement in 6MWD after an EECP treatment and a patient with an improved walking distance by ≥10% was classified as an EECP responder.

All data were collected as a part of clinical routine care during the treatment period. Ethical approval was given by the Swedish Ethical Review Authority in Stockholm (no. 2016/1276–31). The study protocol conforms to the ethical guidelines of the Declaration of Helsinki.^28^

### 2.3 Statistical analysis

Statistical analysis was carried out using IBM SPSS Statistics version 21. Descriptive statistics were used to compute data regarding baseline characteristics. All data are shown as mean ± SD or median and range as appropriate or in the case of categorical variables as n (%). Between‐group difference were analyzed using unpaired *t*‐test in parametric variables and with the X^2^ or Fisher's exact test as appropriate in dichotomous variables. Initially, theoretically driven (i.e., traditional cardiovascular risk factors like age, sex, hypertension, diabetes mellitus, and dyslipidemia)^29^ and clinically relevant baseline characteristics were analyzed with univariate testing and were chosen to enter the regression model if p‐values were less than .05. Potential independent variables were all entered the model at first through the standard entry method (i.e., at the same time). Thereafter, a multinomial logistic regression analysis with stepwise selection was performed using backward elimination, which involved analysis at each step. Independent variables were either retained or deleted based on their statistical contribution in the regression model. This procedure was repeated until only theoretically sound independent variables remained in the model. The success of the model was analyzed with the chi‐square statistic and likelihood ratio tests. The proportion of the variance in the dependent variable was controlled with Nagelkerke (i.e., equivalent to adjusted *R* ^2^). All independent variables were entered as categorical or dichotomous in the analysis. The statistical significance level was set at p < .05.

## 3 RESULTS

### 3.1 Baseline characteristics

A total of 49 patients (41.2%) were classified as responders to EECP (Table 1). In both the responder and the nonresponder groups, most of the patients were men (77.6 and 84.3%, respectively), but no difference was seen regarding mean age (65.7 and 64.6, respectively). There was no significant between‐group difference even though a higher proportion of patients were married or in a relationship in the responder group (73.5 and 64.2%, respectively) (p = .290). Former smokers, and higher baseline NT‐proBNP were also more common in the responder group but without significant difference. Although, overweight (body max index [BMI] > 25 kg/m^2^) was more prevalent among the nonresponders, the between‐group difference was not statistically significant (p = .077). There was a significantly higher proportion of the patients in the responder group that were in a greater functional impairment depicted by CCS class ≥ 3 compared to the non‐responder group (76.6 and 56.1%, respectively, p = .024). In both groups, over 65% of the patients had a prior myocardial infarction, and comorbidities such as hypertension and hyperlipidemia were also common. Though without statistical difference between the groups, diabetes mellitus was more prevalent in the nonresponder group (p = .113). Nonresponders had undergone revascularization treatment more frequently than responders, but the difference was not significant. Around 39% of patients had undergone both percutaneous coronary intervention (PCI) and coronary artery bypass grafting (CABG). Antianginal medications did not differ between the groups.

**TABLE 1:** Baseline characteristics in patients with refractory angina pectoris categorized as responders or nonresponders. Response was based on at least 10% improved 6 min walking distance (6MWD) after one course of enhanced external counterpulsation (N = 119)

	Response, N = 49 *n* (%)	Nonresponse, N = 70 *n* (%)	p value
	Unless otherwise stated	Unless otherwise stated	
Sociodemographic data			
Gender			
Male	38 (77.6)	59 (84.3)	.352
Women	11 (22.4)	11 (15.7)	
Age			
Mean, (SD)	65.7, (8.7)	64.6, (9.3)	.521
Marital status			
Single/widow/divorced	13 (26.5)	25 (35.7)	.290
Married/partner	36 (73.5)	45 (64.2)	
Smoking habit			
Nonsmoker	13 (26.5)	23 (34.3)	.370
Smoker/former smoker	36 (73.5)	44 (65.7)	
Missing data	–	3	
Clinical data			
Systolic blood pressure, mmHg			
Mean (SD)	130 (15.3)	132 (14.3)	.471
Diastolic blood pressure, mmHg			
Mean, (SD)	75 (8.2)	74 (8.3)	.394
Heart rate, bpm			
Mean, (SD)	69 (9.3)	68 (10.0)	.422
Body mass index, kg/m^2^			
>25	39 (79.6)	63 (94.0)	.077
Missing data	‐	3	
Functional class			
CCS Class ≥ 3	36 (76.6)	37(56.1)	.024^a^
Missing data	2	4	
NT‐proBNP, ng/L			
Mean (SD)	654.8 (1150)	460.33. (793)	.287
Missing data	1	4	
Left ventricular ejection fraction			
> 50%	21 (44.7)	19 (28.4)	.072
Missing data	2	3	
Revascularization			
PCI	34 (69.4)	50 (71.4)	.504
Missing Data	1	1	
CABG	26 (53.1)	43 (61.4)	.235
Both interventions	16 (32.7)	31 (44.3)	.201
No revascularization	5	8	.833
Coronary artery status			
Nonobstructive CAD	3 (6.8)	4 (6.0)	
Diffuse CAD	6 (13.6)	7 (10.6)	
Significant stenosis	24 (54.5)	42 (63.6)	
Not significant stenosis	11 (25.0)	13 (19.6)	
Missing data	5	4	
Comorbidities			
Diabetes mellitus	18 (36.7)	36 (51.4)	.113
Hypertension	32 (65.3)	51 (72.9)	.377
Hyperlipidemia	24 (49.0)	36 (51.4)	.793
Myocardial infarction	33 (67.3)	46 (65.7)	.853
Heart failure	13 (26.5)	16 (22.9)	.646
Renal function			
eGFR < 60.0 ml/min	12 (24.5)	13 (18.6)	.322
Missing data	9	9	
Pulmonary disease/asthma	5 (10.2)	8 (11.4)	.833
Peripheral vascular disease or bypass	3 (6.1)	5 (7.1)	.568
EECP‐related data			
D/S ratio, average week 1			
≥ 1.0	24 (49.0)	34 (48.6)	.556
Pharmacologic treatments			
Lipid‐lowering agents	47 (95.9)	64 (91.4)	.284
Beta blockers	43 (87.8)	55 (78.6)	.196
Antiplatelet agents	41 (83.7)	63 (90.0)	.306
Long‐acting nitrates	41 (83.7)	54 (77.1)	.382
Calcium channel blockers	28 (57.1)	39 (55.7)	.877
Diuretics	23 (46.9)	22 (31.4)	.086
Angiotensin receptor blockers	24 (49.0)	24 (34.3)	.108
Angiotensin‐converting‐enzyme‐inhibitors	17 (34.7)	27 (38.6)	.666
Anticoagulants	9 (18.4)	12 (17.1)	.863

Abbreviations: CABG, coronary artery bypass grafting; CAD, coronary artery disease; CCS, Canadian Cardiovascular Society classification; D/S ratio, the peak diastolic to systolic pressure ratio; EECP, enhanced external counterpulsation; eGFR, estimated glomerular filtration rate; NT‐proBNP, N‐terminal of the prohormone brain natriuretic peptide; PCI, percutaneous coronary intervention.

^a^ Statistically significant.

### 3.2 Predictors of clinical response to EECP

Univariate testing with independent theoretically driven variables (i.e., commonly used in cardiovascular research such as comorbidities like myocardial infarction, diabetes mellitus, and heart failure) did not predict response to EECP treatment except the baseline functional class (p *=* .026) (Table 2). Univariate testing of independent variables like LVEF <50%, and BMI > 25 kg/m^2^ showed a trend toward a positive association. Therefore, these potential variables including functional class, age, and gender together with the most common comorbidities were chosen to be entered the regression model. Further on, even though other potential clinical variables (e.g., marital status, smoking status, prior revascularization, and NT‐proBNP) did not show any statistical between‐group difference or positive univariate testing but based on the difference in proportion (Tables 1 and 2), these independent variables were selected as well to enter the regression model.

**TABLE 2:** Univariate analysis with traditional independent theoretically driven and clinical variables to predict response to enhanced external counterpulsation treatment (binary logistic regression analysis) (N = 119)

Characteristics	Univariate analysis	
	OR (95% CI)	p value
Age	1.01(.97–1.10)	.518
Gender	1.55 (.61–3.94)	.354
Marital status	1.53 (.69–3.43)	.292
Myocardial infarction	1.07 (.50–2.34)	.853
Diabetes mellitus	.55 (.26–1.16)	.115
Hypertension	0.70 (.32–1.55)	.378
Heart failure	1.22 (.52–2.84)	.646
LVEF, <50%	2.04 (.93–4.46)	.074
Functional class, CCS class ≥ 3	2.57 (1.11–5.89)	.026^b^
Body mass index, > 25 kg/m^2^	.38 (.12–1.14)	.085
Nonsmoker	.69 (.31–1.55)	.371
Prior revascularization, ≤ 1 type of intervention^a^	1.64 (.77–3.51)	.203
NT‐proBNP, ng/L	1.0 (1.00–1.00)	.302
D/S ratio, ≥ 1.0	1.02 (.490–2.11)	.965

Abbreviations: CCS, Canadian Cardiovascular Society classification; CI, Confidence interval; D/S ratio, the peak diastolic to systolic pressure ratio; LVEF, left ventricular ejection fraction; NT‐proBNP, N‐terminal of the prohormone brain natriuretic peptide; OR, odds ratio.

^a^ Intervention with percutaneous coronary intervention or coronary artery bypass grafting.

^b^ Statistically significant.

Fourteen independent variables (predictors) were entered into the regression model. Addition of the predictors to a model that contained only the intercept significantly improved the fit of the model (X^2^ (3) = 12.920, Nagelkerke *R* ^2^ = .164, p = .005). In the final reduced model (Table 3) patients with a greater baseline functional impairment (OR 3.10, 95% CI 1.12–8.57), an objective systolic LV dysfunction (OR 2.82, 95% CI 1.02–7.80), and unsuitable for further revascularization (i.e., defined as not treated by PCI or CABG, or treated only with one type of intervention) (OR 2.77, 95% CI 1.06–7.20) had around three times higher odds to be a responder to EECP (p < .05). The reduced model successfully predicted 77.3% of the responder group and 52.7% of the nonresponder group. Overall, 63.6% of the predications were accurate.

**TABLE 3:** The final reduced model of independent variables that predict response to enhanced external counterpulsation treatment (multinomial logistic regression analysis) (N = 99)

Characteristics	Multivariate analysis	
	OR (95% CI)	p value
Functional class, CCS class ≥ 3	3.10 (1.12–8.57)	.029
LVEF, < 50%	2.82 (1.02–7.80)	.045
Prior revascularization, ≤1 type of intervention^a^	2.77 (1.06–7.20)	.037

Abbreviations: CCS, Canadian Cardiovascular Society classification; CI, Confidence interval; LVEF, left ventricular ejection fraction; OR, odds ratio.

^a^ Intervention with percutaneous coronary intervention or coronary artery bypass grafting.

## 4 DISCUSSION

### 4.1 Discussion of the findings

Our study showed that a markedly impaired functional status as depicted by CCS class, an objective evidence of systolic LV dysfunction, and prior performed revascularization (i.e., ≤ 1 type of intervention) were predictors of response to EECP treatment in patients with RAP. Similar results can be seen in Lawson et al., who found that baseline CCS class could predict a 1‐year benefit after completion of EECP.^30^ Similarly, Erdling et al.^15^ reported that EECP treatment was most beneficial in patients suffering from severe angina (i.e., CCS 3–4), resulting in a sustained improvement 2 years after completion of treatment. Compared to these two studies, our study differs in design in terms of outcome measure. However, the choice of using an objective measure in our study can be considered a strength since an objective measurement is independent of the observer. Although CCS classification is well known, it is dependent on the observing healthcare professional. Lawson et al.^30^ found that the absence of heart failure was predictive of treatment benefit, but we found that evidence of systolic LV dysfunction was a predictor for response. Although systolic LV dysfunction is not always synonymous with clinical heart failure, many patients with RAP present with systolic LV dysfunction as many of these patients have previously suffered a myocardial infarction, where systolic LV dysfunction is a common complication.^31^, ^32^ Moreover, our results indicate that EECP is a clinically feasible alternative for alleviating symptom burden, especially in patients with a greater functional impairment. Patients with RAP are often seen as “no option patients,” and this view can imply a significant burden on the patient's disease perception and on the healthcare professionals' attitude toward pursing new treatment modalities.^4^ Interestingly, no other study has reported that prior revascularization influences improvement in physical capacity after EECP. Our findings showed that patients who were unsuitable for repeated revascularization with different types of coronary interventions were more likely to be responders. This might be explained by the fact that these patients might suffer from less complicated RAP (e.g., no multiple vessel disease). This growing patient population needs healthcare professionals that embrace treatment modalities beyond the exiting conventional treatments. A sizable proportion of patients have unsuitable anatomy, diffuse distal CAD or a huge burden of comorbidities that make further interventional approaches inapplicable. A significant proportion of patients suffer also from nonobstructive CAD.^33^ Many in this subgroup have shown to have coronary microvascular dysfunction, which is associated with a higher rate of major adverse cardiovascular events.^33^ Identifying microvascular dysfunction may represents a therapeutic target of unmet needs.^3^, ^33^, ^34^ It is suggested that EECP provides positive effects through mechanisms such as improvement of endothelial function and coronary perfusion and flow, which might offer considerable benefits by alleviating symptoms to these patients as well.^34^ Surprisingly, in the present study many traditional risk factors, baseline characteristics, and comorbidities did not predict response. For example, baseline D/S ratios did not predict response, which might be explained by the fact that individual pattern of how the D/S ratio change during treatment is not showed by a mean value collected during the first week. Sahlen et al.^18^ showed that level of NT‐proBNP predicted response to EECP treatment. Another study validated a novel four‐factor models' ability to predict 5 year risk of secondary cardiovascular events in patients with CAD.^29^ Novel risk factors including NT‐proBNP, high‐sensitive cardiac troponin T, and urinary albumin: creatinine ratio were superior to traditional risk factors, except for current smoking.^29^ Future studies are needed to explore whether it will be necessary to use such novel risk factors to identify responders to EECP.

Since regular physical activity is protective and has many benefits,^35^ it will be important to improve the physical capacity in patients with RAP as many of these patients previously have had a sedentary lifestyle. Since EECP treatment is not widely available in Europe, identifying potential responders is reasonable to ensure that the treatment can be preferentially offered to this subgroup. Increased 6MWD after EECP can be one clinically relevant parameter to demonstrate an improved physical capacity, which is a likely surrogate for a better well‐being. Several studies have shown that EECP treatment has the potential to influence a patient's willingness and capability to engage in physical activity.^9^, ^19^, ^36^ Furthermore, improving physical capacity not only could affect HRQoL but also increase the opportunity to achieve guideline recommendations regarding physical activity.^3^

Improvement in the care management, promotion of medication adherence and behavioral counseling could probably be best accomplished by combining a multidisciplinary team and a person‐centered care approach. However, behavioral changes are challenging and to initiate and maintain healthy behaviors is a major mission in the care management of CAD.^37^ A recent study has shown that EECP treatment can significantly reduce level of cardiac anxiety among patients with RAP. However, even if anxiety is decreased, residual cardiac anxiety can lead to avoidance of physical activities.^19^ A potential effect of regular contact with a cardiac nurse considering the EECP treatment regime might have an influence on cardiac anxiety or have a placebo effect of the treatment, which can be a potential confounder that cannot be adjusted. Healthcare professionals are therefore encouraged to use counseling interventions to promote physical activity.

### 4.2 Limitations

This study has a few limitations that need to be considered. First, it is an observational study and residual confounding can be a concern. The choice of using the standard entry method and backward elimination was considered appropriate as the study deals with a small set of predictors and limited knowledge of which independent variables create the best prediction equation. The data were retrieved from a real‐world population of EECP‐treated patients with only one on‐going EECP‐device at time, which, based on the open design, lack of comparison group, and low number of participants limited comparisons and analyses. Considering our long period of patient enrolment, change of staff and conditions could have been a problem. However, three out of five EECP‐educated cardiac nurses have remained in the clinic since the beginning of the study. Consequently, since only a few cardiac nurses have been conducting the 6MWTs and following the same guidance, the possible influence on patients' performance of different operators is deemed to be relatively small. Another aspect was that 6% of the study sample had nonobstructive coronary arteries and it could be reasonable to investigate these patients further for microvascular dysfunction. Due to lack of proper equipment at the study site during the study period, we are unable to present these data. No clear‐cut recommendation regarding cut‐off value of 6MWD was another limitation. Different cut‐offs have been considered such as change of 50 meters, or improvement by 5 or 10%. However, our choice of a 10% increase in 6MWD was deemed reasonable based on the intention to create a distinct difference between responders and nonresponders without limiting the options for statistical comparisons. Finally, our choice of outcome variable decreased the possibility to compare our result with other studies in this research area as previous studies mostly used a clinical measure such as CCS class.

## 5 CONCLUSIONS

EECP treatment should be considered for patients with RAP who have a greater functional impairment, objective evidence of systolic LV dysfunction, and exposure to fewer types of revascularization, either PCI or CABG. Increase in 6MWD after EECP could imply improvement in physical capacity, which is a likely contributor to a better well‐being for patients who are otherwise suffering from limiting symptoms. Identification of potential responders to EECP treatment before initiation of treatment is considered important as the treatment is not widely available and is relatively time‐consuming. Increased knowledge about patients who most likely will respond to treatment opens the possibility that this group should preferentially be prescribed EECP by a cardiologist.

## CONFLICTS OF INTEREST

The authors declare that there is no conflict of interest.

## Data Availability

The data that support the findings of this study are available on request from the corresponding author. The data are not publicly available due to privacy or ethical restrictions.
